# Added value of ultra-short echo time and fast field echo using restricted echo-spacing MR imaging in the assessment of the osseous cervical spine

**DOI:** 10.1007/s11547-023-01589-7

**Published:** 2023-01-13

**Authors:** Eva Deininger-Czermak, Dominic Gascho, Sabine Franckenberg, Pascal Kälin, Christian Blüthgen, Christina Villefort, Michael J. Thali, Roman Guggenberger

**Affiliations:** 1 Department of Forensic Medicine and Imaging, Institute of Forensic Medicine, University of Zurich, Zurich, Switzerland; 2 Institute of Diagnostic and Interventional Radiology, University Hospital Zurich, Raemistrasse 100, 8091 Zurich, Switzerland; 3 Orthopedic Surgery, Balgrist University Hospital, Zurich, Switzerland

**Keywords:** Ultra-short echo time, Restricted echo-spacing, Bone imaging, Degenerative cervical spine

## Abstract

**Purpose:**

To evaluate the added value of ultra-short echo time (UTE) and fast field echo resembling a CT using restricted echo-spacing (FRACTURE) MR sequences in the assessment of the osseous cervical spine using CT as reference.

**Materials and methods:**

Twenty-seven subjects underwent postmortem CT and MRI within 48 h. Datasets were anonymized and analyzed retrospectively by two radiologists. Morphological cervical spine alterations were rated on CT, UTE and FRACTURE images. Afterward, neural foraminal stenosis was graded on standard MR and again after viewing additional UTE/FRACTURE sequences. To evaluate interreader and intermodality reliability, intra-class correlation coefficients (ICC) and for stenosis grading Wilcoxon-matched-pairs testing with multiple comparison correction were calculated.

**Results:**

Moderate interreader reliability (ICC = 0.48–0.71) was observed concerning morphological findings on all modalities. Intermodality reliability was good between modalities regarding degenerative vertebral and joint alterations (ICC = 0.69–0.91). Compared to CT neural stenosis grades were more often considered as nonsignificant on all analyzed MR sequences. Neural stenosis grading scores differed also significantly between specific bone imaging sequences, UTE and FRACTURE, to standard MR sequences. However, no significant difference was observed between UTE and FRACTURE sequences.

**Conclusion:**

Compared to CT as reference, UTE or FRACTURE sequence added to standard MR sequences can deliver comparable information on osseous cervical spine status. Both led to changes in clinically significant stenosis gradings when added to standard MR, mainly reducing the severity of neural foramina stenosis.

## Introduction

Degenerative changes of the spine trigger substantial disease burden, show an increasing prevalence with age and will become a widespread health issue as life expectancy increases [1]. In the cervical spine, symptoms include severe neck pain, radiculopathies and neurological deficits as degenerative foraminal stenosis leads to compression of the radicular nerve exiting through the foramen on its way to target tissues [2, 3]. For an early and correct diagnosis, performing CT and MRI examinations together provide adequate image contrast and resolution: while the first offers optimal bone assessment, the latter allows better visualization of soft tissues, including those involved in nerve entrapment. In order to avoid broad scale use of ionizing radiation from CT, MRI with increased bone depiction quality would be optimal for comprehensive cervical spine assessment, especially in patients suffering from neural compression symptoms [4].

The challenge in bone visualization on MRI is manifold. It bases on the extremely short T2-relaxation time of osseous tissue because of few and tightly bound water molecules [5]. Hence, specific, respectively, modified sequences have been developed in the last decades. Ultra-short echo time (UTE) MR sequences with echo times (TE) of about 0.1 ms allow to partially compensate for the generally low T2w-signal in bones and ligaments [6, 7]. After applying two radiofrequency impulses, a fast radial read out of the *k*-space can be provided, and thus, the induced signal can be measured before the signal of the osseous tissue drops to zero. Until now UTE sequences have already been investigated to evaluate bony, cartilaginous or ligamentous structures, focusing on the implementation in a clinical setup [8–11]. Beside UTE sequences, other technical approaches have been developed and investigated, such as “Slab-selective UTE” applying a modified radio frequency pulse, or zero-echo time (ZTE) sequences turning on readout gradients even before the radio frequency pulse. [12–14].

Another option to better depict bone on MRI is a 3D optimized multi-echo sequence, presented in the literature as fast field echo resembling a CT using restricted echo-spacing (FRACTURE) sequence [15, 16]. Here, numerous gradient echoes are acquired in a fixed time interval, summed up in the end and get subtracted from the last echo. The summation of all echoes leads to a high T2-weighted signal and the subtraction of this sum from the last echo generates a CT-like image [16, 17]. The common obstacle, especially in a clinical setting, regarding bone imaging MR sequences is the factor of time. Therefore, only few studies were able to compare new MR sequences to CT as reference standard within a short time interval between scans to provide an optimal comparison.

In this postmortem study, we investigated the depiction quality of the osseous cervical spine provided by UTE and FRACTURE MR sequences compared to a CT reference standard. In addition, the influence of adding specific MR bone imaging to routinely use standard MR sequences on neural foraminal stenosis grading was assessed.

## Material and methods

All 27 subjects (16 males, 11 females) with a median age of 73 years (range: 44–93 years) underwent postmortem CT and postmortem MRI examinations within 48 h.

### CT imaging

CT scans were performed on a 128-multi-slice CT scanner (Somatom Definition Flash, Siemens Healthineers, Forchheim, Germany). To optimize visualization, we adapted the scan protocol according postmortem scan recommendations using a tube voltage of 120 kVp, tube current of 1000 mAs, resulting in a volume-weighted CT dose index of 91 mGy [18]. The image dataset was reconstructed with a slice thickness of 0.6 mm and an increment of 0.4 mm using a bone kernel (H60) and a soft kernel (H31). A maximum field of view of 300 × 300 mm^2^ with a matrix of 512 × 512 was used.

### MRI imaging

Neck MRI was conducted on a 3 Tesla MR scanner (Achieva 3.0 TX, Philips Healthcare, Best, the Netherlands) using a 16-channel head and neck coil. In addition to standard MR sequences, UTE and FRACTURE sequences for dedicated bone imaging were acquired. All detailed scan parameters are shown in Table 1.

**Table 1:** Detailed MR scan parameters

Sequence	TR (ms)	TE (ms)	Flip angle	Scan time (min:sec)
T1wTSE	556.4	8.0	90°	03:20
3D T2wTSE	2000	234.0	90°	08:00
SPIR	2620.0	90	90°	04:42
UTE	0.2	10.2	10°	12:34
FRACTURE	4.6	20.7	15°	07:24

#### Standard sequences

The following sequences were acquired in sagittal plane with a field of view of 230 × 230 × 119 mm and a slice thickness of 3 mm: a T1-weighted turbo spin echo (TSE) sequence, a 3D T2-weighted TSE sequence and a T2-weighted TSE sequence with spectral presaturation with inversion recovery (SPIR).

#### UTE sequence

Images were acquired in sagittal planes with a voxel size of 0.8 × 0.8 × 1.2 mm^3^ using a field of view of 230 × 230 × 119 mm^3^. The flip angle was 10°. No specific post-processing was conducted.

#### FRACTURE sequence

The 3D FRACTURE sequence was acquired with four in-phase echoes, isotropic voxels (voxel size: 0.7 × 0.7 × 0.7 mm^3^) and a field of view of 230 × 230 × 182 mm.^3^. Echoes were acquired every 4.6 ms to ensure that fat and water were in phase. Images from shorter TEs helped to increase signal-to-noise ratio (SNR), whereas the longest TE image, which resembled a T2-weighted image, improved image contrast [17]

### Image analysis

First, all images were anonymized and for each modality a dataset was created (CT, UTE, FRACTURE and standard MR sequences). Every dataset contained the same anonymized cases, but in a randomized order. First, degenerative changes of three parts of the cervical spine (upper C2–C4, middle C4–C6 and lower part C6–TH1) had to be rated independently, on CT, then on UTE and FRACTURE images. An interval of at least 2 weeks was demanded between the readouts.

All qualitative readouts were conducted by two independent radiologists (5 and 7 years of experience), who were blinded to each other using Syngo.via (version 30A_HF91, Siemens, Healthcare). As scoring system, a 4-point Likert scale was used to describe qualitative criteria (0—none, 1—mild, 2—moderate, 3—severe). Both readers were free to change and/or invert the gray scales on all images if desired. The following qualitative image findings associated with boney spine changes were scored: anterior and posterior hyperostosis, endplate sclerosis, degeneration of facet, uncovertebral and costovertebral joints (left/right), anterior and posterior spondylolisthesis and spinal canal narrowing [19, 20].

To facilitate multi-parameter comparisons and calculations, all rated criteria were separated into summated scores for morphology: productive changes, including hyperostosis, endplate sclerosis or erosions, degeneration load of all joints and spinal canal stenosis including spondylolisthesis and spinal canal narrowing.

**Fig. 1:**
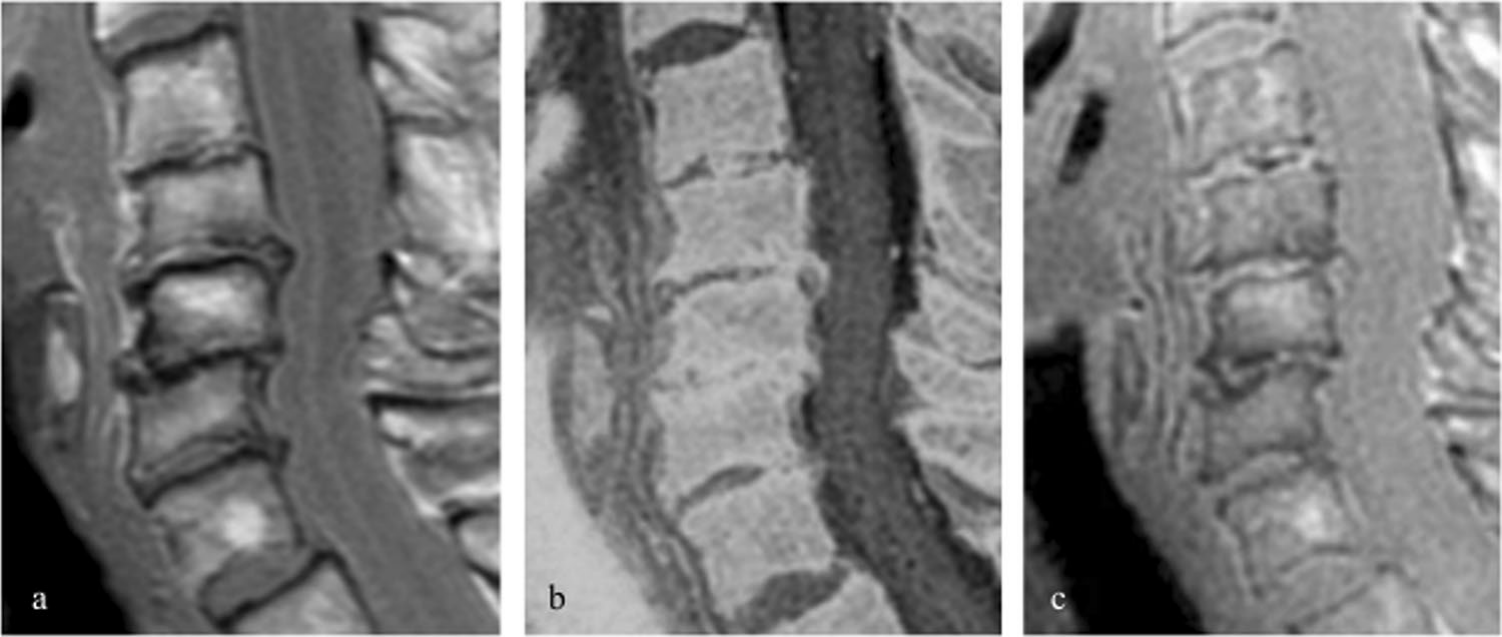
Imaging of the cervical spine: Exemplary images in sagittal plane on standard T1-weighted TSE (**a**), FRACTURE (**b**) and UTE (**c**) sequence of a degenerative altered cervical spine. Note significant productive changes with posterior spondylophytes, endplate sclerosis and slight retrolisthesis in mid-cervical segments

In the second readout neural foraminal stenosis, based on the perineural fat obliteration, in the most severely degenerated mid-cervical segments (C3–C6) were assessed [21, 22]. Because every segment had to be assessed individually, the total amount of graded segments was 27 × 3 = 81 (Fig. 1). Bilateral stenosis grades of 81 × 2 = 162 neural foramen were rated by both readers, resulting in 2 × 162 = 324 individual ratings. To simulate a standard clinical scenario and to test for a potential incremental value of UTE/FRACTURE images, first only standard MR sequences were rated. Afterward, UTE and FRACTURE images were provided, and score changes were documented. In addition, neural foraminal stenosis were scored on CT images with a time interval to the MR readout of at least one month. Stenosis grades rated as moderate or severe were considered as clinically significant, mild or none as nonsignificant.

To quantify SNR and contrast to noise ratio (CNR) of UTE and FRACTURE images, regions of interest (ROI) with a size of 5 mm^2^ were drawn by one reader in following anatomical areas: base of C4, base C5, left and right semispinal muscle. The noise level was defined as the standard deviation (SD) of the measured ROI in the pharynx containing air. SNR and CNR were calculated with routinely used formula: SNR = SI_bone_/SD_air_, CNR = (SI_bone_–SI_muscle_)/SD_air_.

### Statistical analysis

For statistical analysis SPSS, version 26.0 (IBM, Armonk, New York) was used. A *p* value of < 0.05 was considered significant. Interreader and intermodality reliability was calculated by performing a two-way mixed model, and for all parametric values, the intra-class coefficient (ICC) with a confidence interval of 95%, based on the terminology of McGraw and Wong [23], was applied. ICC values less than 0.5 were considered poor, between 0.5 and 0.75 moderate, between 0.75 and 0.9 good, and greater than 0.9 excellent agreement [24].

To legitimate nonparametric test, a Gaussian distribution was first excluded by applying a Shapiro–Wilk test. For further evaluation of the added value of UTE and FRACTURE sequences to standard sequences, a Wilcoxon-matched pairs test was used. To correct for multiple comparison in case of significant *p* values, a Holm–Bonferroni test was conducted post hoc with an alpha level of 0.05 [25]. For comparisons regarding SNR and CNR, a *t* test was applied.

## Results

### Degeneration of the osseous cervical spine

**Fig. 2:**
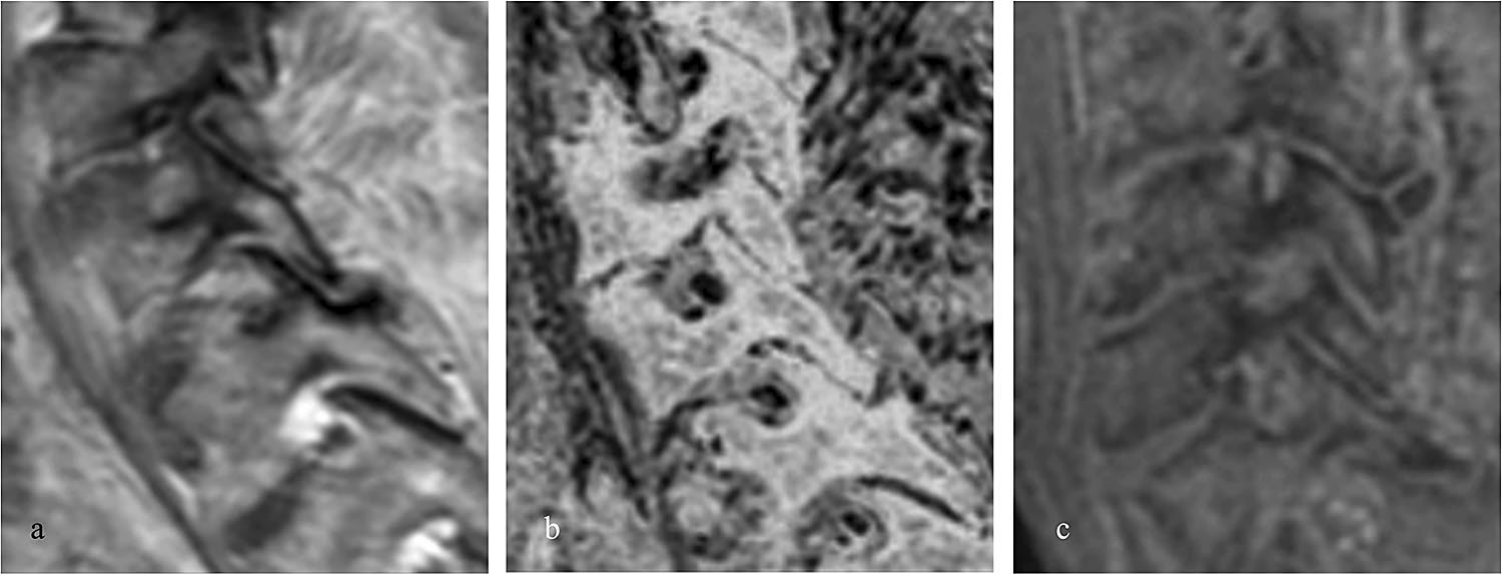
Images of neural foramina on T1-weighted TSE (**a**), FRACTURE (**b**) and UTE (**c**). Especially on UTE stenosis grading varied between readers, resulting in divergent changes of initially made diagnosis on standard MR sequences. On FRACTURE, both readers tended to upgrade stenosis gradings

In total, 81 segments were rated by each reader (Fig. 2). An overview of all scores is shown in Table 2.

**Table 2:** Qualitative ratings of all reader regarding morphological features of the cervical spine

	UTE		FRACTURE		CT	
	Reader 1	Reader 2	Reader 1	Reader 2	Reader 1	Reader 2
*Upper part*						
Productive score	2.4 (1.2)	1.4 (1.8)	2.9 (0.8)	1.9 (2.1)	2.7 (1.7)	2.0 (2.3)
Joint degeneration	4.2 (1.0)	5.9 (2.7)	5.1 (1.5)	6.1 (2.6)	5.0 (1.9)	5.6 (3.2)
Spinal canal stenosis	0.3 (0.7)	0.6 (0.8)	1.0 (1.4)	0.4 (1.1)	0.6 (1.3)	0.4 (1.0)
*Middle part*						
Productive score	4.2 (1.4)	3.6 (2.1)	4.0 (1.3)	4.0 (2.5)	4.0 (1.8)	4.1 (2.3)
Joint degeneration	4.7 (1.6)	6.7 (1.7)	6.6 (2.2)	6.7 (2.6)	6.1 (2.5)	6.2 (2.8)
Spinal canal stenosis	0.8 (1.3)	1.0 (1.1)	1.3 (1.4)	0.6 (1.0)	0.9 (1.2)	0.7 (0.9)
*Lower part*						
Productive score	3.2 (1.2)	2.5 (2.2)	3.7 (1.0)	3.0 (2.5)	3.2 (1.5)	3.0 (2.3)
Joint degeneration	4.2 (1.1)	5.6 (2.2)	6.1 (2.0)	5.7 (2.8)	5.1 (2.2)	5.2 (2.3)
Spinal canal stenosis	0.4 (0.7)	0.5 (0.8)	0.6 (0.6)	0.3 (0.8)	0.5 (0.8)	0.3 (0.7)

Mean and standard deviation in parenthesis of morphological features are shown for both readers in detail in relation to the upper, middle and lower part of the cervical spine. The maximum points for the productive score are 9 points (0 none, < 3 mild, 3–6 moderate, > 6 severe), for the joint degeneration score 12 points (0 none, < 4 mild, 4–8 moderate, > 8 severe) and for the spinal canal stenosis score 6 points (0 none, < 2 mild, 2–4 moderate, > 4 severe)

Interreader reliability showed overall the best results on CT with a moderate agreement for the productive and joint degeneration scores (ICC = 0.70, CI: 0.53–0.81) and moderate agreement for the spinal canal stenosis score (ICC = 0.67, CI: 0.48–0.79). UTE ratings showed overall less agreement, especially for joint degeneration scores (ICC = 0.48, CI: 0.19–0.67), whereas FRACTURE ratings showed the highest reliability for joint degeneration scores (ICC = 0.73, CI: 0.58–0.83) (Table 3).

**Table 3:** Overview of interreader and intermodality reliabilities

	Productive score	Joint degeneration	Spinal canal stenosis
*Interreader correlation*			
CT	0.71 (0.55–0.81)	0.70 (0.53–0.81)	0.67 (0.48–0.79)
UTE	0.70 (0.53–0.81)	0.48 (0.19–0.67)	0.54 (0.29–0.71)
FRACTURE	0.69 (0.52–0.80)	0.73 (0.58–0.83)	0.48 (0.19–0.66)
*Intermodality correlation*			
CT to UTE to FRACTURE	0.92 (0.89–0.91)	0.84 (0.79–0.88)	0.78 (0.71–0.83)
CT to UTE	0.88 (0.84–0.91)	0.80 (0.73–0.86)	0.69 (0.58–0.76)
CT to FRACTURE	0.88 (0.83–0.91)	0.78 (0.69–0.84)	0.77 (0.68–0.83)

Interreader and intermodality correlation coefficient in details with the confidence intervals in parenthesis. The productive score was rated most consistent on all modalities and between the readers

Concerning productive changes, intermodality variability, comparing both MR sequences to CT reference standard resulted in a good agreement between CT and UTE (ICC = 0.88, CI: 0.84–0.91), as well as CT and FRACTURE (ICC = 0.88, CI: 0.83–0.91). For joint degeneration scores, the correlation coefficient showed slightly lower, but still good agreement between CT and UTE (ICC = 0.80, CI: 0.73–0.86) as well as CT and FRACTURE (ICC = 0.78, CI: 0.69–0.84). Ratings of the spinal canal narrowing were less reliable between all modalities with only moderate to good agreement (Table 2).

### Neural foraminal stenosis

Overall interreader agreement regarding the grading of neural foramina was moderate (ICC = 0.59; CI: 0.56–0.62). For further intermodality comparison, gradings of the senior reader were analyzed. As described above, neural foraminal stenosis was distinguished between significant and nonsignificant. Therefore, stenosis grades could be upgraded from nonsignificant to significant and vice versa. On the initial CT, 110 (67.9%) were rated nonsignificantly, whereas 52 (32.1%) were considered significantly narrowed. Comparing CT to standard MR sequences, 72.2% of neural foramina were rated equal, 19.8% foramina were graded less severe on MR, and only 8.0% were rated higher.

Adding UTE sequence to standard MRI sequences resulted in an upgrade from nonsignificant to significant stenosis in 4.9%, in a downgrade from significant to nonsignificant in 15.4% and equal ratings in 79.6%.

After adding FRACTURE to the standard sequences, ratings were more often downgraded than upgraded. 14.2% were changed to nonsignificant stenosis, whereas only 1.2% were changed to a significant stenosis. The majority (84.6%) were graded equally.

Changes of stenosis gradings were significant different between standard and each bone specific MR sequence (all *p* < 0.05).

Compared to CT, MR stenosis grades, after adding UTE, showed in 65.4% an equal, in 28.4% a lower and in only 6.2% a higher rating. Providing additionally FRACTURE, 69.1% of all foramina showed the same stenosis grading as on CT, 27.8% were downgraded, but only 3.1% upgraded.

Grading of neural foraminal stenosis differed between all modalities, except between FRACTURE and UTE (*p* = 0.21), after applying Holm–Bonferroni multiple comparison correction significantly.

### Quantitative analysis

**Fig. 3:**
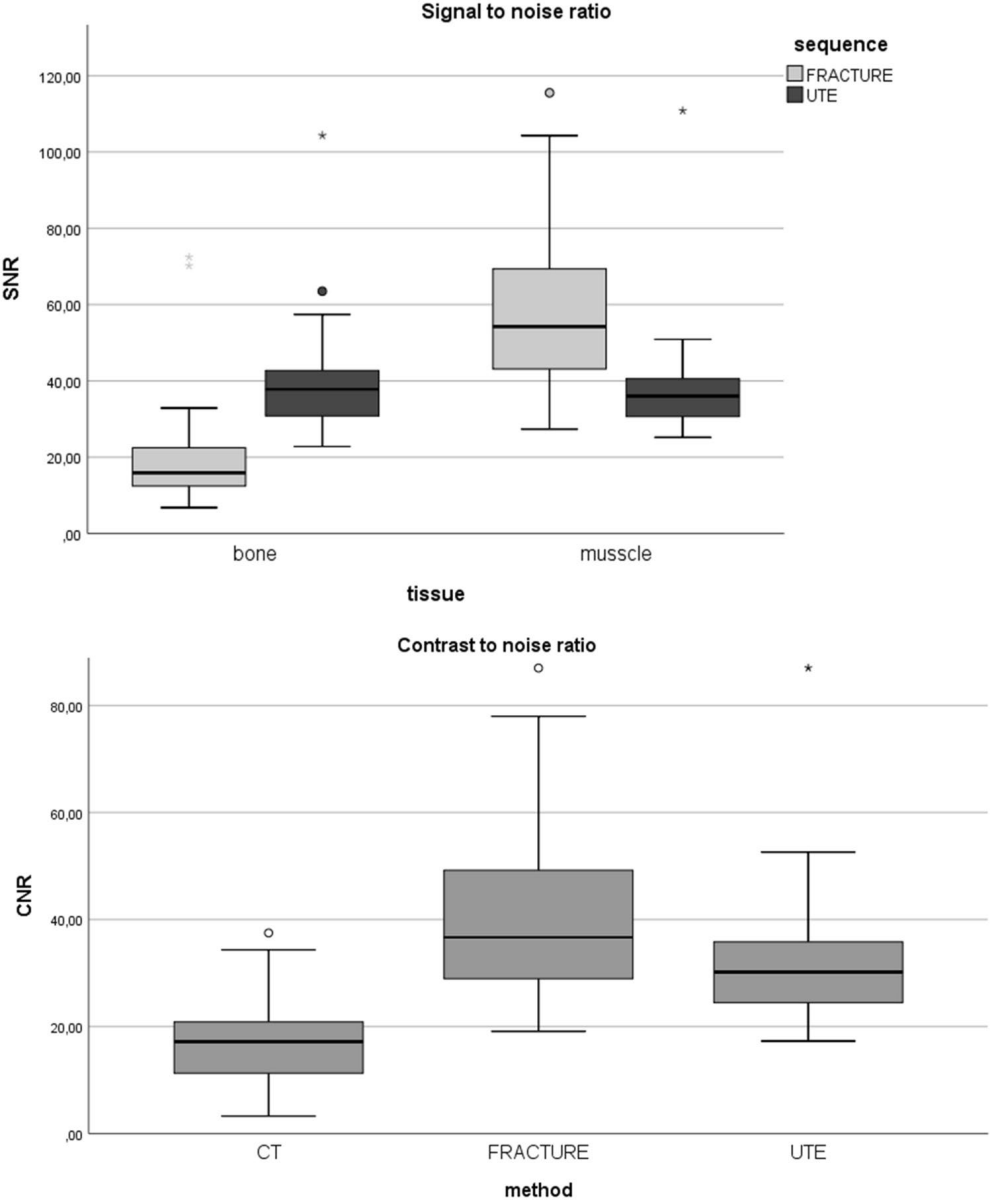
Boxplots of SNR and CNR values, showing the difference between both MR bone imaging sequences. The highest SNR in bone resulted on UTE; however, soft tissue was higher on FRACTURE. Regarding CNR values, considering bone structures to its adjacent soft tissue FRACTURE showed the best CNR compared to UTE and also CT

The mean SNR in soft tissue was highest on FRACTURE with a significant difference to UTE images; however, SNR in bone was significantly higher on UTE than on FRACTURE. The mean CNR, regarding the contrast between bone and soft tissue, was highest on FRACTURE sequences compared to both other imaging methods (both *p* < 0.05) (Fig. 3).

## Discussion

We evaluated the potential value of MR sequences optimized for bone visualization in the assessment of the cervical spine and neural foraminal stenosis.

To grade as objectively as possible osseous degenerative changes, we used CT as a reference standard for comparison of osseous architecture. In the length of C2 to TH1, we observed variable degrees of degeneration, with more severe alterations in the lower cervical spine, which correlates to numerous previous studies [26, 27]. Regarding morphological criteria, represented by productive, joint degeneration as well as spinal canal stenosis scores, ICC was highest on CT, which confirms its role as reference standard regarding osseous structures. Interestingly, we observed variable correlations within MR sequences comparing morphological criteria, which is not in accordance to previously published articles which focused on interreader agreements in the cervical spine on standard sequences and zero-echo time sequences [28, 29]. On UTE, joint degeneration scores showed lower ICC, whereas the productive score showed a similar agreement to CT. This may be due to the sagittal plane acquisition or/and the limited access to this relatively new sequence. On FRACTURE MR images, only the spinal canal stenosis score indicated less agreement between all readers compared to the other two scores which were comparable to CT. Isotropic voxels of the FRACTURE sequence provide an advantage over UTE sequence, and therefore, especially the joint degeneration score, can be assessed easier. Another advantage of FRACTURE sequence is a certain preservation of surrounding tissue signal, which is basically eliminated using UTE technique. Nevertheless, while interreader agreements differed depending on the modality, ratings of each reader differed only slightly between modalities. Therefore, the good intermodality correlation can be considered as an indicator that all modalities are appropriate to assess osseous changes.

The diagnosis of significant neural foraminal stenosis is important in clinical routine as a degenerative stenosis as a cause of severe pain and radiculopathies may require targeted periradicular infiltration or surgical release [30, 31]. We evaluated if the diagnosis of nonsignificant and significant stenosis changes compared to standard sequences, after additional MR sequences were provided. Overall, adding UTE sequence to standard MR images significantly less neural foramina were considered significantly stenosed, although the majority was rated equal. Regarding FRACTURE sequence, it showed similar rating changes as more foraminal stenosis grades were downgraded than upgraded. However, whether UTE or FRACTURE was added, only 20% of all grading scores were changed in either direction. This may be an indicator that both sequences led readers rather to confirm their initial rating and strengthen their diagnosis. To compare MR to CT ratings, neural foramina were also rated on CT. According to previous conducted studies, a good comparability between CT and MR was stated [32, 33]. Our results are partly conformant with these previously published results. Only about 70% of neural foraminal stenosis were rated equally to CT on our routinely used sequences. This was lower than in previous studies, which also investigated neural foraminal stenosis using routinely used sequences [32]. However, considering studies which focused on not-routinely used sequences such as ZTE sequences, which are not applicable at all standard MR hardware, our results show less conformity regarding neural foramina comparability between bone imaging sequences and CT [29, 32]. Neural foramina were mainly downgraded to nonsignificant stenosis. Interestingly, there was a no significant difference between UTE and FRACTURE images considering the severity of stenosis. Therefore, an equal performance of both added sequences was shown. The probability to downgrade a significant stenosis to a nonsignificant stenosis on FRACTURE or UTE compared to CT is highest; however, if a stenosis is not significant on CT, it is most probably confirmed on FRACTURE or UTE images.

To quantify signal and contrast performance of the bone imaging sequences, we calculated SNR and CNR. Here, we observed the highest SNR and the highest CNR was obtained on FRACTURE images. The high CNR between muscle and bone on FRACTURE may be due to the summation of all echoes, providing a higher contrast between osseous and soft tissue structures.

Regarding new MR sequences, including UTE and FRACTURE, scan time needs to be addressed. To optimize image quality, scan time in this study was not optimized. Because of the postmortem setting this was no issue, however, in a clinical setting this is a major factor in conducting an examination. Considering clinical implementation, previous studies have shown that UTE sequences are adaptable for clinical routine scan times and are tolerable for patients [34, 35].

One limitation of this study was the postmortem setting; however, we only included subjects with a short postmortem interval to avoid any impaired image quality by postmortem changes such as gas formation. Moreover, because of stable tissue characteristics of bone, postmortem changes are much later present than in soft tissue. In previous MR studies regarding bone imaging, comparable image quality was observed in postmortem and in-vivo cases [8]. We also did not include any cases with traumatic cervical spine injuries; therefore, we cannot conclude the performance of the bone imaging sequences for those.

### Conclusion

Adding UTE or FRACTURE sequence to standard MR sequences can deliver comparable information on osseous cervical spine status when compared to CT. Both led to changes in clinically significant stenosis gradings on standard MR sequences, mainly reducing the severity of neural foramina stenosis.

This research did not receive any specific grant from funding agencies in the public, commercial or not-for-profit sectors.
